# Development of Gas-Chromatographic Method for Simultaneous Determination of Cannabinoids and Terpenes in Hemp

**DOI:** 10.3390/molecules25245872

**Published:** 2020-12-11

**Authors:** Jure Zekič, Mitja Križman

**Affiliations:** 1 Department of Food Chemistry, National Institute of Chemistry, Hajdrihova 19, SI-1000 Ljubljana, Slovenia; jure.zekic@ki.si; 2 Faculty of Chemistry and Chemical Technology, University of Ljubljana, Večna pot 113, SI-1000 Ljubljana, Slovenia

**Keywords:** cannabinoids, terpenes, cannabis, hemp, gas chromatography, capillary column

## Abstract

An original gas chromatographic method has been developed for simultaneous determination of major terpenes and cannabinoids in plant samples and their extracts. The main issues to be addressed were the large differences in polarity and volatility between both groups of analytes, but also the need for an exhaustive decarboxylation of cannabinoid acidic forms. Sample preparation was minimised, also by avoiding any analyte derivatisation. Acetone was found to be the most appropriate extraction solvent. Successful chromatographic separation was achieved by using a medium polarity column. Limits of detection ranged from 120 to 260 ng/mL for terpenes and from 660 to 860 ng/mL for cannabinoids. Parallel testing proved the results for cannabinoids are comparable to those obtained from established HPLC methods. Despite very large differences in concentrations between both analyte groups, a linear range between 1 and 100 µg/mL for terpenes and between 10 and 1500 µg/mL for cannabinoids was determined.

## 1. Introduction

The hemp plant (*Cannabis sativa* and *Cannabis indica*), or simply cannabis, is a plant that has excited much interest throughout history because of its characteristics and various possibilities of use. The popularity of cannabis has increased especially over the last few years, as its widespread usefulness, including use for medical purposes, is becoming increasingly noticeable [1,2,3,4,5]. Hemp is known to contain various groups of compounds, probably the most characteristic among them are cannabinoids. Furthermore, cannabis also contains diverse terpenes, flavonoids, and some other groups of compounds [6,7,8,9].

Cannabinoids are probably the most studied metabolites of cannabis. Many of their beneficial effects on human health are already known, and there is also a lot of ongoing research, discovering new ones [10]. As a result, the use of cannabinoids in a wide variety of preparations is growing, which is also reflected in increased cannabis production. At the same time, a need for an efficient routine analytical method for monitoring the cannabinoid content in plant material has arisen. A number of methods for the analysis of cannabinoids in cannabis have indeed already been developed; among various approaches, the predominant is chromatographic analysis, in particular using gas chromatography (GC) [11,12,13,14,15,16,17,18] or high performance liquid chromatography (HPLC) [16,19,20,21,22,23,24,25,26,27].

Even though gas chromatography used to be the most common technique for analysis of cannabinoids in cannabis extracts, HPLC is currently increasingly gaining popularity in this field of application. HPLC determination of cannabinoids, in comparison to the analysis with GC, has some significant advantages: above all, it avoids the potential aggravating circumstances caused by the high temperature of analysis at GC, which affects the results mainly during the phase of sample injection and also indirectly during the analysis itself. Cannabinoids are found mainly in acidic forms in the plant, which eventually decarboxylate if they are exposed to raised temperature [28]. The temperature in the gas chromatograph also causes the process of decarboxylation, which is reflected in the results in two ways: we cannot separately determine acidic and decarboxylated forms of a particular cannabinoid, but only their total content. On the other hand, there is a significant probability that decarboxylation in the injector will not proceed completely [29]. Especially at higher cannabinoid concentrations, this may be reflected in apparently lower values measured and consequently irregular results of analysis. Both problems can be successfully solved by the derivatisation of cannabinoids (including their acid forms) in the sample [17,30,31,32]. However, this represents an additional step that is often not desirable, because it increases probability for experimental error and prolongs analysis time, which may be a considerable drawback in terms of method suitability for routine use. With HPLC, all of these problems have been successfully avoided, as some relatively rapid, simple, and effective methods for the determination of both acidic and decarboxylated cannabinoids in cannabis samples have already been developed [16,19,20,21,22,23,24,25,26,27,33].

Thus, two major approaches to chromatographic analysis of cannabinoids in hemp most often appear in the literature; direct analysis of a suitably diluted sample extract by liquid chromatography [16,19,20,21,22,23,24,25,26,27,33], or preliminary derivatisation of the extract and subsequent analysis by gas chromatography [17,30,31,32]. Despite its mentioned drawbacks, the latter approach is still quite in use, somewhat for traditional reasons, but also for entirely practical reasons, since GC instrumentation is simpler and less expensive than HPLC and sometimes, consequently, more economical for use or maybe even the only one available. 

For direct gas chromatographic analysis of cannabinoids, traditionally, the most commonly used stationary phase is 5% phenyl 95% dimethylpolysiloxane [7,16,18], followed by 100% dimethylpolysiloxane phase [11,13]. Recently, more polar stationary phases like 35% phenyl 65% dimethylpolysiloxane have also been used, with a potential gain in selectivity [34,35].

Another relatively important group of compounds in cannabis are terpenes [6,7,8,9]. Different varieties of cannabis contain mainly different monoterpenes and sesquiterpenes, which also give a distinctive scent to hemp plants. From an analytical point of view, they are interesting because their profile is often characteristic of a particular variety or population of cannabis, which may enable identification of different plant specimens [36,37]. Terpenes in cannabis are also often credited for the so-called “entourage” effect [37]. The analysis of terpenes is most often performed using gas chromatography as a separate type of analysis; successful separation and determination on different types of columns is usually quite fast, effective and simple. 

An unavoidable step in chromatographic analysis of plant material is analyte extraction from the sample. Both groups of compounds, terpenes and cannabinoids, can be extracted from the plant material by different approaches. For cannabinoids, the most common is classical extraction with a relatively apolar solvent (usually ethanol) either by mechanical shaking or by ultrasonic extraction. On the other hand, GC analysis of terpenes can also be done by the headspace sampling technique [38]. The alternative to headspace sampling is of course solvent extraction, in such a case a solvent of appropriate polarity must be chosen in relation to the analytes of interest. Terpenes and cannabinoids differ both in terms of volatility and polarity, as well as in the concentrations found in hemp samples. Terpene levels are usually significantly lower compared to cannabinoids. As demonstrated by Namdar et al. [7], an optimum solvent for terpene extraction was found to be a mixture of ethanol:hexane (3:7, *v*/*v*), while for cannabinoids they corroborated the use of ethanol as the optimum solvent.

Therefore, a simultaneous analysis of terpenes and cannabinoids in hemp samples is not a trivial task. A notable example of such a type of methodology has been published by Franchina et al. [39]. In this case, the methodology involved the use of sorptive extraction and thermal desorption sampling, two-dimensional gas chromatography, and mass detection. Such a methodology is certainly very detailed and useful when advanced studies have to be done, like in chemotaxonomy. For routine analyses, however, such a setup is probably too complicated and expensive. The aim of the presented work was to find appropriate conditions, mainly in terms of sample preparation, for a simultaneous analysis of both groups of compounds, while keeping the overall experimental and instrumental setups simple.

## 2. Results and Discussion

### 2.1. Sample Preparation

As already exposed in the introduction, the main challenge in combined analysis of terpenes and cannabinoids was the sample preparation step and more precisely, the extraction conditions. As already pointed out by Namdar et al. [7], optimum solvent composition for terpenes and cannabinoids differs. During their work, they found the mixture of ethanol:hexane (3:7, *v*/*v*) to be the best compromise for extracting both groups of compounds. During the present work, the quest for a single solvent similar in properties to the mentioned work was undertaken. The solvent selection was then narrowed to acetone and ethyl acetate, also because they are regarded as solvents with low environmental impact [40]. Finally, acetone was selected as the most appropriate solvent, based on the extraction recoveries obtained.

Another important parameter highly affecting the results is the sample-to-volume ratio during extraction. Besides having a direct effect on extraction efficiency as well, this ratio also has implications on the final analyte concentrations. Good overall analyte recoveries were obtained with sample-to volume ratios between 1:10 and 1:25. During method development, it was found that a ratio of about 1:17 (i.e., 300 mg per 5 mL of solvent) was a good compromise between extraction efficiency and still providing sufficient concentrations of terpenes in working sample solutions in order to be quantified without concentrating the solution. Terpenes are unfortunately very volatile and significant analyte losses can be expected with any of the solvent evaporation techniques [7]. Therefore, this sample preparation step was deliberately avoided. At the same time, cannabinoid concentrations in sample solutions proved to be below the upper practical limit in terms of detector linearity. Compared to terpenes, cannabinoids are more problematic to analyse. Besides their lower volatility, the possibility of incomplete decarboxylation of cannabinoid acidic forms during sample vaporisation and injection must be prevented. This phenomenon is more pronounced at higher concentrations [29].

In practice, the concentrations of major cannabinoids in the extracts were kept at up to 1.5 mg/mL or below. By using such high cannabinoid concentrations, it was less challenging to quantify minor cannabinoids as well.

### 2.2. Gas Chromatographic Separation

**Figure 1:**
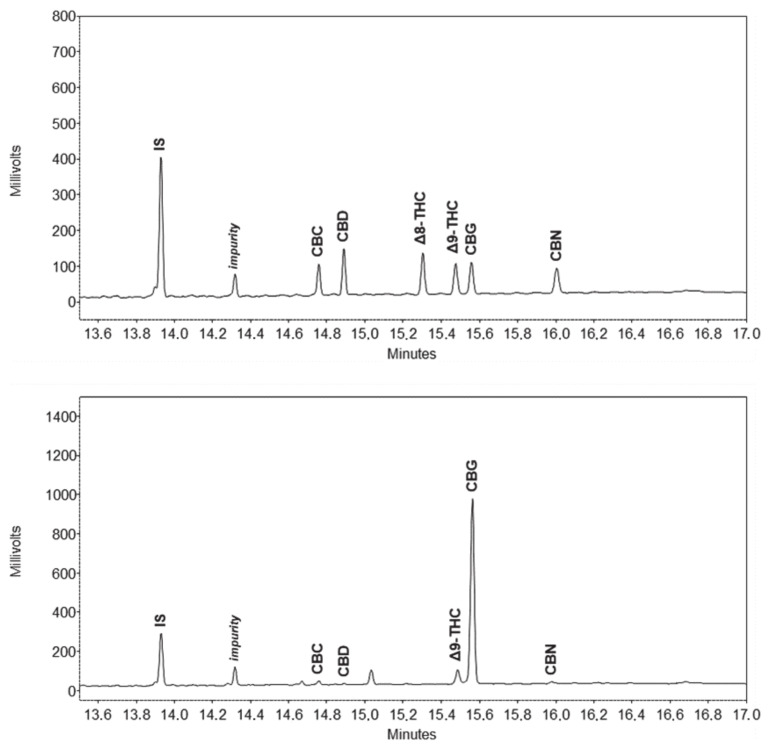
Chromatograms (displayed tR = 13.5–17.0 min) of cannabinoid standard solution (**top**) and hemp plant (cannabigerol (CBG) chemotype) extract (**bottom**). Peak labelling: IS—internal standard, CBC—cannabichromene, CBD—cannabidiol, Δ8-THC—Δ8-tetrahydrocannabinol, Δ9-THC—Δ9-tetrahydrocannabinol, CBG—cannabigerol, CBN—cannabinol.

**Figure 2:**
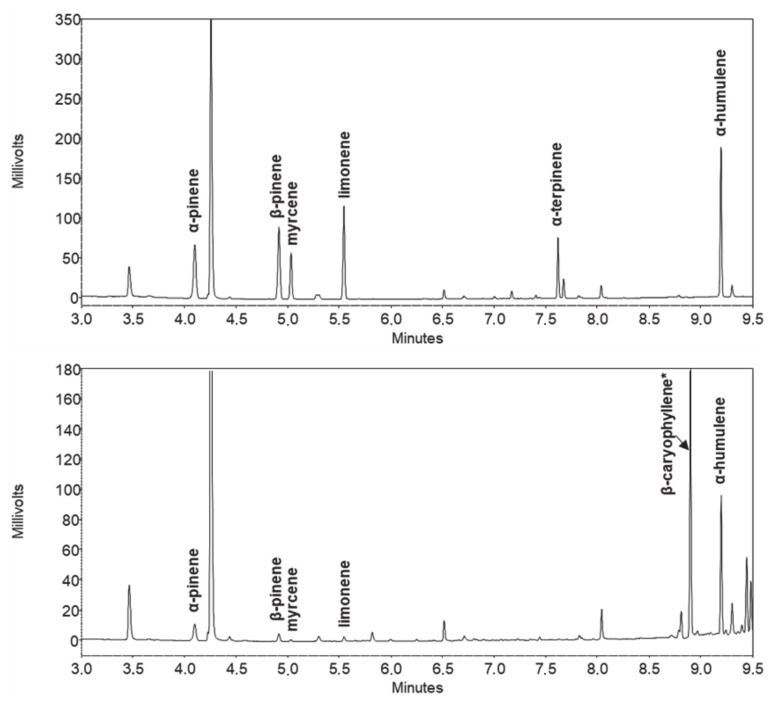
Chromatograms (displayed tR = 3.0–9.5 min) of terpene standard solution (**top**) and hemp plant extract (**bottom**). *β-caryophyllene was identified by mass-spectrometric data.

**Figure 3:**
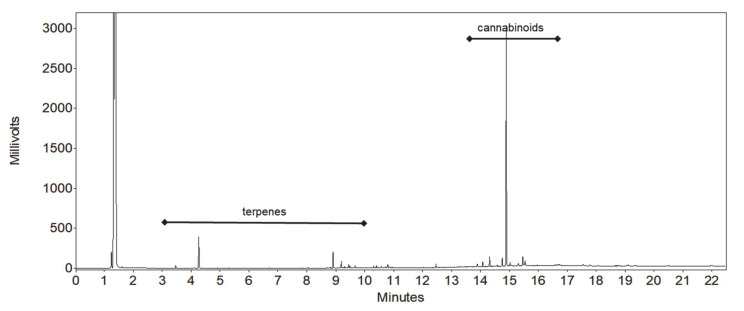
Chromatogram of hemp sample extract (full scale).

Terpenes and cannabinoids differ widely, both in terms of polarity and volatility. These two groups of compounds are therefore easily separated between each other using GC, although a wide temperature gradient program is needed due to a large difference in volatility. Successful separation of individual terpenes is not particularly challenging, as many works demonstrate [7,8,36,38,41,42,43]. The main challenge was to provide good separation of some cannabinoids, the most critical being the resolution between cannabichromene (CBC) and cannabidiol (CBD). Using the ubiquitous stationary phase based on 5% phenyl 95% dimethylpolysiloxane, the resolution between those two peaks proved to be unsuitable, since these two peaks overlapped, as proven by preliminary tests (data not published). Much better results are obtained using more polar stationary phases like 35% phenyl 65% dimethylpolysiloxane, as recent applications also demonstrate [34,35]. As a consequence, the choice for an even more polar stationary phase was made, namely 50% phenyl 50% dimethylpolysiloxane. According to initial expectations, a good resolution was obtained, and no issues related to overlapping of cannabinoid peaks was observed anymore. At the same time, using a relatively polar stationary phase did not impair the separation of terpenes. In fact, even more polar stationary phases were employed for terpenes [41,42,43]. Chromatograms of standard solutions and sample extracts are depicted on Figure 1, Figure 2 and Figure 3.

### 2.3. Method Performance and Validation

The developed method exhibited good overall analytical performance within a relatively short analysis time, since it provided separation of two major groups of compounds in the cannabis plant. The most difficult to separate, CBD and CBC, were fully baseline resolved. Other analytes were also separated with excellent resolution. Validation and stability data (Table 1) confirmed the suitability of the method also for quantitative use. Sufficiently low sensitivity limits, which are important especially from the terpenes standpoint, and on the other hand, accurate results also for higher cannabinoid concentrations, allow work with non-concentrated or non-diluted working sample solutions. This is a great advantage especially in view of the process simplicity.

### 2.4. Comparison with HPLC

In order to confirm correctness of the results obtained from the newly developed GC method, HPLC analysis was performed, applying a previously published and validated method [33]. As mentioned before, GC analysis may not be sufficiently accurate mainly at higher cannabinoid concentrations. Therefore, HPLC quantification was performed for the two major cannabinoids in the samples—CBD or CBG—and their acidic forms which occur in rather high concentrations. The total content of these cannabinoids, measured by HPLC analysis, was in very high accordance with results obtained from GC analysis (Table 2). This also directly confirms the correctness of the developed GC method in this respect. Aside from being inherently a less sensitive methodology than HPLC [33], as depicted in Table 1, GC cannabinoid analysis is performance-wise fully comparable to HPLC. For most analytes, better repeatability figures were also obtained.

## 3. Materials and Methods 

### 3.1. Chemicals, Reagents and Samples

P.a. grade ethyl acetate and n-hexane from Merck (Darmstadt, Germany) and acetone from Honeywell (Seelze, Germany) were used for standards preparation and sample treatment. HPLC grade methanol from Honeywell (Seelze, Germany) and deionised water from a Milli-Q apparatus (Millipore, Milford, MA, USA) were used to prepare mobile phase for HPLC analysis. Ammonium formate was LC–MS grade (Sigma-Aldrich, St. Louis, MO, USA).

Terpene reference standards of α-humulene (96%), limonene (97%), myrcene (>90%), α-pinene (99%), β-pinene (97%), and α-terpinene (95%) were obtained from Sigma-Aldrich (St. Louis, MO, USA). 

Cannabinoid reference standards of cannabigerol (CBG, 99%) and cannabidiol (CBD, 99.9%) in solid form were obtained from LGC standards (Teddington, Middlesex, UK). Δ8-tetrahydrocannabinol (Δ8-THC), Δ9-tetrahydrocannabinol (Δ9-THC), cannabichromene (CBC) and cannabinol (CBN) were obtained as solution in methanol (1 mg/mL) from LGC standards as well.

Squalane (96%) was purchased from Sigma-Aldrich (St. Louis, MO, USA) and was applied as internal standard (IS).

Buds of industrial hemp plant (stemless) material of various cultivars were obtained from local hemp growers.

### 3.2. GC Analysis

Analysis was performed by a Focus GC with FID detector (Thermo Scientific, Rodano, Milan, Italy) with a Rtx-50 capillary column (30 m × 0.25 mm × 0.25 µm film thickness) (Restek Corporation, Bellefonte, U.S.A.). One microlitre of sample was injected with a split ratio 30:1 at 310 °C using ultrapure grade helium as carrier gas at 2 mL/min. The GC oven temperature program started at 60 °C (3 min), followed by a linear gradient of 20 °C/min to 290 °C, temperature was then kept constant for 8 min. Flame ionisation detector temperature was 310 °C.

Thermo Electron Trace 2000 GC coupled with Thermo Electron DSQ quadrupole mass spectrometer (Thermo Scientific, Rodano, Milan, Italy) with an electron ionisation source at 70 eV in positive mode was applied for the purpose of terpene identification. Some parameters had to be adapted for GC-MS analysis. Namely, 0.5 µL of sample was injected with a split ratio of 60:1, flow rate of carrier gas was 1 mL/min. Other parameters remained the same as for GC-FID analysis. The MS data were acquired in full scan mode from *m*/*z* 50–450 with acquisition frequency of 4.2 scans per second.

### 3.3. HPLC Analysis

Analysis was performed according to the published method [33]. For HPLC analysis, sample solutions (as described in Section 3.5) were further diluted with methanol 100-fold.

### 3.4. Standard Solutions Preparation

Stock solutions of solid cannabinoid standards (CBD and CBG) were prepared in acetone in the concentration of 2.0 mg/mL. Working standard solutions of CBD and CBG were prepared in the concentration range of 0.05 to 1.5 mg/mL, and working standard solutions of CBC, CBN, Δ8-THC, and Δ9-THC were prepared in the concentration range of 0.01 to 0.2 mg/mL. All working solutions contained internal standard of concentration 0.13 mg/mL and were prepared by diluting stock solution with acetone.

Stock solutions of terpene standards were prepared in the concentration of 2.0 mg/mL in acetone, except for myrcene stock solution, which was prepared in hexane. Working standard solutions consisted of a mix of all terpene standards in the concentration range of 1.0 to 100 µg/mL.

### 3.5. Samples and Preparation of Extracts

Dried and powdered plant materials (300 mg) were extracted by sonication for 30 min at room temperature with acetone or ethyl acetate containing IS (130 µg/mL). Sample solutions were then centrifuged at 16.000g for 10 min and the supernatant was transferred into GC vials. 

### 3.6. Quantitation, Method Precision, Accuracy, Sensitivity, Linearity, and Stability

Injection precision was determined by five injections of working standard solution. Extraction efficiency was assessed by three consecutive extractions of selected plant samples and then comparing the analyte recovery with the combined recovery of all extraction steps. Accuracy was determined by spiking sample solution with cannabinoid standards at concentration of 0.25 mg/mL and terpene standards at concentration of 0.025 mg/mL. Repeatability and intermediate precision were also tested on a homogeneous plant sample. Three replicates were assayed for repeatability, while three replicates were assayed on each of the 3 consecutive days for intermediate precision.

Linearity was checked in the range of standard solutions concentration (0.05 to 1.5 mg/mL for CBD and CBG; 0.01 to 0.2 mg/mL for CBC, CBN, Δ8-THC, and Δ9-THC; and 0.001 to 0.1 mg/mL for terpenes). Correlation coefficients were calculated with intercept values set at zero. Analyte peak areas were normalised by dividing them with IS peak areas. For stability tests, a sample solution was refrigerated at 4 °C in the dark for 48 h.

In order to confirm the correctness of the results, HPLC analysis of properly diluted sample extracts solutions in methanol was performed.

## 4. Conclusions

A GC-FID method for simultaneous analysis of terpenes and cannabinoids in hemp samples has been developed. The main issues concerning the method were ensuring appropriate sample preparation conditions for both terpenes and cannabinoids, successful separation of critical cannabinoid peaks, and quantitative decarboxylation of cannabinoid acidic forms. Acetone proved to be an appropriate solvent for quantitative extraction of all the analytes concerned, which occur in a wide concentration range, using a sufficiently low sample-to-solvent ratio. Separation-wise, the resolution between CBD and CBC peaks was substantially improved by using a relatively polar column with 50% phenyl 50% dimethylpolysiloxane stationary phase. Quantitative decarboxylation was ensured using a high injector temperature and low injection volumes. Peak identity was confirmed by GC-MS. Despite many of the method parameters being near the practical limits in terms of instrumentation capacity like temperature, detector response, etc., it provides a robust tool for simultaneous quantitative analysis of these two chemically different groups of analytes.

## Figures and Tables

**Table 1:** Method validation parameters.

Analyte	Injection Precision (% RSD, *n* = 5)	Accuracy (%)	Extraction Efficiency (%)	Repeatability (% RSD, *n* = 3)	Intermediate Precision (% RSD, *n* = 9)	LOD ^b^ (µg/mL)	LOQ ^b^ (µg/mL)	Regression Coefficient (*R*)	Stability 48 h (%)
CBD	0.17 (1.27)	100.3 (97.3)	92.0	0.08 (0.78)	1.59 (1.27)	0.662 (0.093)	2.207 (0.310)	0.9999 (1.0000)	99.9 (102.7)
CBG	1.60 (0.52)	97.6 (96.9)	92.1	0.96 (2.00)	1.77 (2.48)	0.697 (0.94)	2.322 (0.313)	0.9999 (0.9999)	98.2 (101.2)
Δ^8^-THC	0.62 (1.51)	99.1 (93.7)	- ^a^	0.89 (0.94)	2.21 (2.53)	0.817 (0.205)	2.724 (0.684)	0.9999 (0.9999)	99.4 (95.6)
Δ^9^-THC	0.54 (0.65)	98.8 (93.4)	93.1	0.42 (0.85)	2.35 (2.34)	0.822 (0.196)	2.739 (0.654)	1.0000 (0.9999)	99.6 (94.0)
CBC	0.79 (0.18)	100.1 (113.3)	92.0	1.34 (6.53)	1.67 (9.02)	0.815 (0.024)	2.716 (0.082)	1.0000 (0.9986)	98.0 (103.9)
CBN	1.27 (0.20)	98.9 (88.4)	- ^a^	1.25 (3.38)	1.88 (3.86)	0.857 (0.007)	2.858 (0.023)	0.9995 (0.9999)	100.7 (103.8)
α-pinene	1.34	99.6	95.5	0.54	1.52	0.259	0.862	0.9999	99.8
β-pinene	0.65	100.1	95.2	1.39	1.33	0.175	0.585	0.9999	97.7
mircene	1.79	100.2	- ^a^	0.65	3.15	0.183	0.609	0.9998	100.7
limonene	1.05	99.9	95.9	0.64	2.89	0.124	0.412	0.9999	100.3
α-terpinene	0.62	100.2	- ^a^	1.54	2.83	0.193	0.642	1.0000	97.0
α-humulene	0.75	100.8	93.2	0.75	1.60	0.185	0.616	0.9999	99.6

Analyte abbreviations are as referred in the text. LOD—limit of detection, LOQ—limit of quantification. ^a^ Second consecutive extraction of samples gave no detectable peaks for the analyte. ^b^ Determination of LOD and LOQ was based on extrapolation of signal-to-noise responses. Data in parentheses are validation parameters obtained with our HPLC method [33] for comparison purposes (where applicable).

**Table 2:** GC and HPLC method comparison.

Hemp Sample Cultivar	Fedora 17	Carmagnola	Futura 75	Santhica
Sample Solution	1	2	3	4
CBDA (µg/mL)—HPLC	298	1110	-	-
CBD (µg/mL)—HPLC	485	468	-	-
CBGA (µg/mL)—HPLC	-	-	428	510
CBG (µg/mL)—HPLC	-	-	118	106
HPLC—total CBD *	783	1578	-	-
HPLC—total CBG *	-	-	546	616
GC—total CBD	839	1635	-	-
GC—total CBG	-	-	605	572
CBD—relative difference (%)	+7.1	+3.6	-	-
CBG—relative difference (%)	-	-	+10.8	−7.1

* total CDB/CBG are calculated as equimolar equivalents expressed in decarboxylated forms.
